# Poly(ethylene glycol)s as grinding additives in the mechanochemical preparation of highly functionalized 3,5-disubstituted hydantoins

**DOI:** 10.3762/bjoc.13.3

**Published:** 2017-01-04

**Authors:** Andrea Mascitti, Massimiliano Lupacchini, Ruben Guerra, Ilya Taydakov, Lucia Tonucci, Nicola d’Alessandro, Frederic Lamaty, Jean Martinez, Evelina Colacino

**Affiliations:** 1 Department of Engineering and Geology (INGEO), G.d’Annunzio University of Chieti-Pescara, Via dei Vestini, 31, 66100 Chieti Scalo, Italy; 2 Université de Montpellier, Institut des Biomolécules Max Mousseron (IBMM), UMR 5247 CNRS - UM - ENSCM, Place E. Bataillon, Campus Triolet, 34095 Montpellier CEDEX 5, France; 3 P.N. Lebedev Institute of Physics of RAS, Leninskiy pr-t, 53, 119991, Moscow, Russia; 4 Moscow Institute of Physics and Technology, Institutskiy per., 9, 141700, Dolgoprudny, Russia; 5 Department of Philosophical, Educational and Economic Sciences, G. d’Annunzio University of Chieti-Pescara, Via dei Vestini, 31, 66100 Chieti Scalo, Italy

**Keywords:** ball-milling, 1,1’-carbonyldiimidazole (CDI), hydantoins, mechanochemistry, liquid-assisted grinding (LAG), poly(ethylene) glycols (PEGs)

## Abstract

The mechanochemical preparation of highly functionalized 3,5-disubstituted hydantoins was investigated in the presence of various poly(ethylene) glycols (PEGs), as safe grinding assisting agents (liquid-assisted grinding, LAG). A comparative study under dry-grinding conditions was also performed. The results showed that the cyclization reaction was influenced by the amount of the PEG grinding agents. In general, cleaner reaction profiles were observed in the presence of PEGs, compared to dry-grinding procedures.

## Introduction

Poly(ethylene) glycols (PEGs) are eco-friendly solvents [1–2], finding applications in the biomedical field and for pharmaceutical formulations [3] and catalysis [4]. PEG-based reaction media [1] are safe reaction environments, efficiently heated by microwaves [5], but their use in organic transformations activated by other alternative energy inputs is still scarce. Only three examples highlight their peculiar role for metal-catalysed processes in a ball mill (Mirozoki–Heck reaction) [6], by ultrasound (copper-catalysed cyanation reaction) [7], and for co-crystal formation in the polymer-assisted grinding process (POLAG) [8]. However, to the best of our knowledge, the systematic investigation of the influence of PEG polymers has not been reported yet for organic syntheses promoted by mechanical energy.

**Scheme 1:**
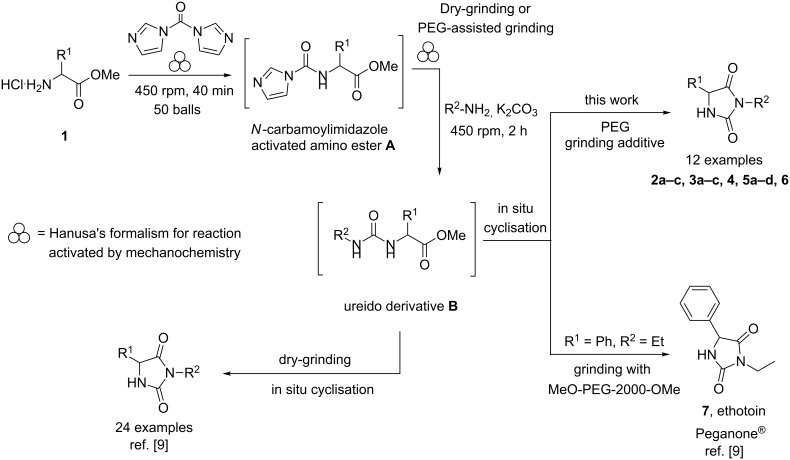
PEG-assisted grinding strategy for the preparation of 3,5-disubstituted hydantoins.

We firstly reported the positive influence of PEG solvents as grinding agents for the mechanochemical preparation of an active pharmaceutical ingredient (API), the anticonvulsant drug ethotoin **7** [9] (marketed as Peganone^®^, Scheme 1). We describe herein the impact of the addition of variable amounts of PEG, PEG chain length and end terminal groups, for the preparation of diverse 3,5-disubstituted hydantoins from α-amino methyl esters **1**, via an in situ intramolecular cyclization reaction of the ureido derivative **B**, which was obtained from *N*-carbamoylimidazole activated amino ester derivative **A** by reaction with various amines [9–11] (Scheme 1). Hanusa’s formalism was used to represent the reaction activated by mechanochemical energy [12].

The yields, reaction rates and chemoselectivity obtained in the presence of melted PEGs were compared with the results obtained in dry-grinding conditions.

## Results and Discussion

H-Leu-OMe was used as benchmark for the mechanochemical preparation of 3-ethyl-5-isobutylhydantoin (**2a**) (R^1^ = CH_2_CH(CH_3_)_2_ and R^2^ = CH_2_CH_3_, Scheme 1) [9]. The reaction was screened in the presence of various PEG additives (Table 1), by adding variable amounts of PEGs, with different molecular weights (600 < *M*_w_ < 5000 Dalton) or chain end groups (dihydroxy, mono- or dimethyl ether substituents) in the second step (Table 1). The first set of experiments was aimed to determine if the addition of variable amounts of solid MeO-PEG-2000-OMe (Table 1, entries 2–5) or HO-PEG-3400-OH (Table 1, entries 7–10) could impact both the reaction yield and rate, compared to dry-grinding conditions previously reported [9] (Table 1, entry 1). Yields were generally improved in the presence of variable amounts of PEGs (Table 1, entries 2, 3 and 7, 8), starting to decrease when reaching a critical value at 675 mg (Table 1, entries 5 and 10). The substrate conversion remained moderate, the cyclization reaction of the corresponding ureido derivative B-Leu was slowed down and the methyl ester moiety was partially hydrolysed. Indeed, the base activity was increased due to the presence of water in PEG as well as by the PEG crown-ether-like effect [1], chelating the potassium cations.

**Table 1:** Screening of grinding additives using (*L)*-H-Leu-OMe^.^HCl as benchmark for the preparation of compound **2a**.^a^

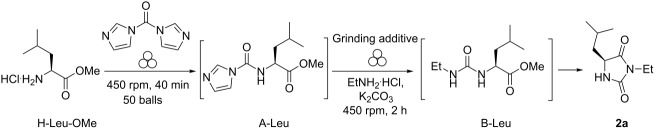			

Entry	Grinding additive	Amount (mg)	Yield (%)^b^

1 [9]	–	–	61
2	MeO-PEG-2000-OMe	225	70
3 [9]		450	70
4^c,d^		450^c,d^	71^c,d^
5		675	29^e^
6	MeO-PEG-2000-OH	450	68
7	HO-PEG-3400-OH	225	77
8		450	73
9^c,d^		450^c,d^	73^c,d^
10		675	66^e^
11	HO-PEG-5000-OH	450	57
12	HO-PEG-1000-OH	450	66
13	HO-PEG-600-OH	450	56
14	Glycerol	450	58

^a^Conditions: (step 1) (*L*)-H-Leu-OMe^.^HCl (1 mmol) and CDI (1.3 equiv.) at 450 rpm, in a planetary ball mill (PBM) using a 12 mL SS jar with 50 balls (SS = stainless steel, 5 mm Ø) for 40 min; (step 2) EtNH_2_^.^HCl (1.6 equiv), K_2_CO_3_ (3.6 equiv) and the grinding additive RO-PEG*_n_*-OR (R = H, Me, *n* = 14, 23, 46, 77, 114) or glycerol (450 mg mmol^−1^) (see Supporting Information File 1 for experimental details); ^b^Isolated yields; ^c^The reaction time in the second step was 3 h; ^d^PEG was precipitated in diethyl ether, then filtered and dried in the air before use [16]; ^e1^H NMR yield.

It is worth noticing here that the crude mixture was cleaner in comparison with dry-grinding conditions. Indeed, the symmetrical urea of the starting amino ester – obtained from the corresponding *N-*carbamoyl imidazole amino ester **A** – was not observed, as shown by the LC–MS analyses of the crude mixture. An approach complementing similar strategies was already described to avoid the formation of symmetrical ureas in solution [13].

The preparation of the hydantoin **2a** was also investigated using batches of solid PEGs (*M*_w_ = 2000 and 3400) in which PEGs with lower molecular weight (*M*_w_ = 200–400) were eliminated before use by a precipitation/filtration procedure (Table 1, entries 4 and 9), according to a well-established protocol [14–16]. Even when the PEG polymers were supposed to be homogeneously liquids (melting point around 55 °C) at the operational temperature, comparable yields could be obtained only by extending the reaction time (3 h instead of 2 h), when ‘pre-treated’ PEGs were used instead of ‘unfiltered’ PEGs (Table 1, entries 3 and 8).

This observation suggested that changes in the ‘physical state of the system could be induced by specific interactions with PEG polymers and influenced both by the viscosity and the polymer chain length. After selecting the optimal polymer amount (450 mg mmol^−1^), the study was carried on by increasing (Table 1, entry 11) or reducing (Table 1, entries 12 and 13) the polymer chain length, changing the end terminal substituents (Table 1, entries 6 vs 3), and adding glycerol instead of PEGs as additive (Table 1, entry 14). As a result, the effect of using different end terminal groups was not markedly significant, the yield was a function of the average molecular weight of the PEG used: HO-PEG-5000-OH (Table 1, entry 11) was probably too viscous to allow the diffusion of reactants. Decreased and comparable yields were also observed by reducing the PEG chain length (Table 1, entry 13) and by using glycerol. It is also worth noting here that not only viscosity, but any modification of the physical state of the system impacted the outcome of the reaction. Indeed, HO-PEG-600-OH (0.119 cSt) led to comparable yields when replaced by a more viscous liquid like glycerol (1.12 cSt) (Table 1, entry 14), an eco-friendly solvent still not investigated for liquid-assisted grinding procedures. In fact such a compound is becoming a green source of several building blocks since glycerol is actually produced in very large amount as byproduct from biodiesel synthesis [17].

With this background, 3-ethyl-5-benzylhydantoin (**3a**) [9] (R^1^ = CH_2_Ph and R^2^ = CH_2_CH_3_) was also prepared using solid MeO-PEG-2000-OMe and HO-PEG-3400-OH as additives (Table 2). Using (L)-H-Phe-OMe^.^HCl as substrate, as a general trend and in comparison with the dry-grinding procedure previously reported [9] (Table 2, entry 1), yields were generally lower with PEG additives, independently on their size and amounts (Table 2). This trend, apparently in contrast with the results illustrated so far for 3-ethyl-5-isobutylhydantoin (**2a**) [9] suggested that the reactivity of the system might be also a function of the nature of the amino ester side chain, influencing the solubility of the reactants, reaction intermediates and final products. However, no differences in yields were observed when (D)-H-Phe-OMe was used, instead of its enantiomer (Table 2, entry 3).

**Table 2:** Optimization of liquid-assisted grinding conditions using (*L*)-H-Phe-OMe^.^HCl as benchmark for the preparation of compound **3a**.^a^

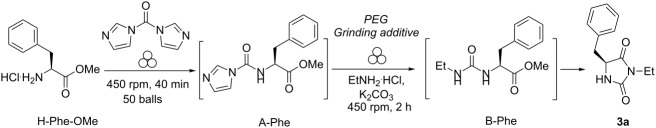			

Entry	Grinding additive	Amount (mg)	Yield (%)^b^ [9]

1 [9]	–	–	84
2	MeO-PEG-2000-OMe	225	59
3^c^		450	70 (68)^c^
4	HO-PEG-3400-OH	225	58
5		450	60
6		675	62

^a^Conditions: (step 1) (L)-H-Phe-OMe^.^HCl (1 mmol) and CDI (1.3 equiv) at 450 rpm, in a planetary ball-mill (PBM) using a 12 mL SS jar with 50 balls (SS = stainless steel, 5 mm Ø) for 40 min; (step 2) EtNH_2_^.^HCl (1.6 equiv), K_2_CO_3_ (3.6 equiv) and RO-PEG*_n_*-OR (R = H, Me, *n* = 46, 77) (see Supporting Information File 1 for experimental details); ^b^isolated yields; ^c^*D*-H-Phe-OMe was used.

Therefore, the one-pot two-steps cyclization reaction was investigated with different amino ester/amine combinations (H-AA-OMe/R^2^-NH_2_) and comparative experiments using dry- or wet-grinding with PEGs (*M*_w_ = 2000 and 3400, 450 mg mmol^−1^) were also performed (Table 3).

**Table 3:** Syntheses of 3,5-disubstituted hydantoins under dry-grinding (conditions A)^a^ or PEG-assisted grinding (conditions B and C).^b^

Entry	H-AA-OMe	Yields (%)^b^ vs conditions^a^			Product

		A	B	C	

1	H-Leu-OMe	61 [9]	70	73	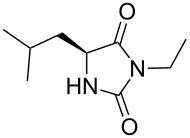 **2a** [9]
2		57 [9]	69	66^c^	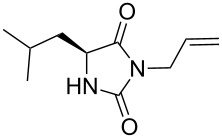 **2b** [9]
3		38 [9]	n.p.^d^	48^c^	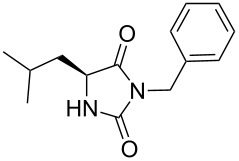 **2c** [9]
4	H-Phe-OMe	84 [9]	70	60	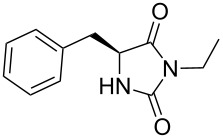 **3a** [9]
5		30	n.d.^e^	n.d.^e^	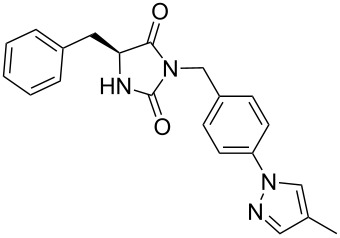 **3b**
6		70	n.d.^e^	n.d.^e^	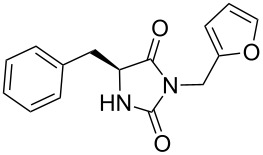 **3c**
7	H-Ser(*Ot-*Bu)-OMe	51 [9]	70	70	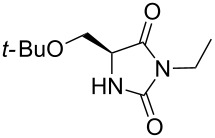 **4**
8	H-Lys(*Z*)-OMe	31 [9]	47^c^	50^c^	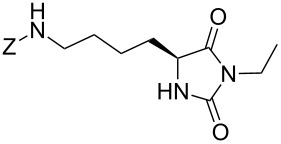 **5a** [9]
9		62	n.p.^d^	37^c^	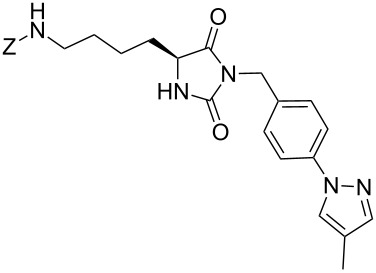 **5b**
10		37	n.p.^d^	n.p.^d^	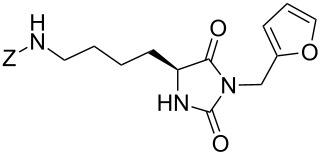 **5c**
11		47	n.p.^d^	n.p.^d^	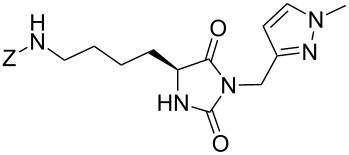 **5d**
12	H-Aib-OMe	46 [9]	62	62	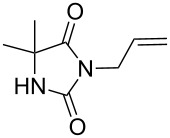 **6** [9]

^a^Conditions: (step 1) *(*L*)-*α-amino ester hydrochloride (1 equiv) and CDI (1.3 equiv) at 450 rpm, in a 12 mL inox jar with 50 balls (stainless steel, 5 mm Ø) for 40 min; (step 2) R^2^NH_2_ (1.6 equiv) and K_2_CO_3_ (3.6 equiv) at 450 rpm for 2 hours. A: the reaction was performed with no additive (dry-grinding); B: MeO-PEG-2000-OMe (450 mg mmol^−1^); C: HO-PEG-3400-OH (450 mg mmol^−1^) were added in the second step (wet-grinding conditions with a PEG additive; ^b^isolated yields; ^c1^H NMR yield on the crude reaction mixture; ^d^the reaction was not performed (n.p.); ^e^the reaction yield was not determined (n.d.).

Indeed, compounds with the same *N-*R^2^ substituent led to variable yields for different amino esters (R^1^, Scheme 1 and Table 3), as shown for experiments performed in both dry-grinding conditions in the series **3b** and **5b** (R^2^ = 1-[4-(4-methyl-1*H*-pyrazol-1-yl)phenyl]methyl, Table 3, entries 5 and 9), **3c** and **5c** (R^2^ = furan-1-ylmethyl, Table 3, entries 6 and 10), and wet-grinding experiments with PEGs, for the series **2a, 3a**, **4** (Table 3, entries 1, 4, and 7, respectively) and **5a** (R^2^ = ethyl, Table 3, entry 8) or **2b** and **6** (R^2^ = allyl , Table 3, entries 2 and 12). However, the PEG influence on the reaction yield could not be excluded. The mechanochemical productivity was slightly improved when PEG polymers were used compared to dry-grinding conditions, as demonstrated for the synthesis of hydantoins **2a–c** (Table 3, entries 1–3), **5a** (Table 3, entry 8) and **6** (Table 3, entry 12), with the exception of hydantoins **3a** (Table 3, entry 4) and **5b** (Table 3, entry 9). Moreover, the preparation of hydantoins **3b** and **3c** (Table 3, entries 5 and 6) in the presence of PEG led to incomplete conversion of starting materials, together with the formation of various unknown byproducts. A possible explanation can be related to the solubility of reactants, reaction intermediates and final products in PEG polymers, although the existence of specific interactions with PEG polymers cannot be excluded. Indeed, especially under mechanical stress, PEGs are known to induce changes in the physical state of the system [8].

These results confirmed the role played by polymers in mechanochemical transformations, also leading to cleaner reaction profiles. However, the choice of the suitable polymer for a specific transformation was not trivial: the ‘fine tuning’ of the physical state of the system was also related to specific physical aspects also connected to the intrinsic properties of the polymer. In addition, PEG polymers were demonstrated as a valid eco-friendly and safe alternative to classic solvents used in liquid-assisted-grinding procedures (LAG) [18–22] due to their low melting point (45–60 °C), enabling their use as melt during grinding, low toxicity and low vapour pressure, reducing the risk of explosions or overpressure that might be encountered on large scale LAG-procedures.

## Supporting Information

File 1 Experimental procedures, characterization of new compounds and copies of ^1^H and ^13^C NMR spectra.
