# Supplementary material for: Poly(ethylene glycol)s as grinding additives in the mechanochemical preparation of highly functionalized 3,5-disubstituted hydantoins
Source: Beilstein J Org Chem. 2017 Jan 4;13:19–25. doi: 10.3762/bjoc.13.3 (PMC5238572; doi:10.3762/bjoc.13.3)
Supplement: File 1 — Experimental procedures, characterization of new compounds and copies of 1H and 13C NMR spectra. [file Beilstein_J_Org_Chem-13-19-s001.pdf]

Supporting Information
for

Poly(ethylene glycol)s as grinding additives in the mechanochemical preparation of highly functionalized 3,5-disubstituted hydantoins

Andrea Mascitti[1,2,‡], Massimiliano Lupacchini[1,2,‡], Ruben Guerra[2], Ilya Taydakov[3,4], Lucia Tonucci[5], Nicola d'Alessandro[1], Frederic Lamaty[2], Jean Martinez[2], and Evelina Colacino*[2]

Address: [1]Department of Engineering and Geology (INGEO), G.d'Annunzio University of Chieti-Pescara, Via dei Vestini, 31, 66100 Chieti Scalo, Italy, [2]Université de Montpellier, Institut des Biomolécules Max Mousseron (IBMM), UMR 5247 CNRS - UM - ENSCM, Place E. Bataillon, Campus Triolet, 34095 Montpellier CEDEX 5, France, [3]P.N. Lebedev Institute of Physics of RAS, Leninskiy pr-t, 53, 119991, Moscow, Russia, [4]Moscow Institute of Physics and Technology, Institutskiy per., 9, 141700, Dolgoprudny, Russia and [5]Department of Philosophical, Educational and Economic Sciences, G. d'Annunzio University of Chieti-Pescara, Via dei Vestini, 31, 66100 Chieti Scalo, Italy

Email: Evelina Colacino* - evelina.colacino@umontpellier.fr

*Corresponding author

‡Equal contributors.

Experimental procedures, characterization of new compounds and copies of ^{1}H and ^{13}C NMR spectra

General remarks and experimental procedures S2–S6
References S6
^{1}H NMR and ^{13}C NMR of compounds **3b,c**, **5b–d** (Table 3) S7–S16

General remarks and experimental procedures

All reagents were commercially available and used without any further purification. *L*-α-amino esters were used, except when otherwise specified. NMR spectra were recorded at room temperature with the appropriate deuterated solvent ($CDCl_3$ or DMSO-d_6). Chemical shifts (*δ*) of ^{1}H NMR and ^{13}C NMR spectra are reported in ppm relative to residual solvent signals ($CHCl_3$ in $CDCl_3$: δ = 7.27 ppm for ^{1}H and $CDCl_3$: δ = 77.04 ppm for ^{13}C NMR; DMSO in d_6-DMSO: δ = 2.54 ppm for ^{1}H and d_6-DMSO: δ = 40.45 ppm for ^{13}C NMR); values for the coupling constants *J* are given in Hz. ^{1}H NMR spectra were registered at 300 MHz and 400 MHz. ^{13}C NMR spectra were registered at 75 MHz and 100 MHz. HRMS measurements were performed on a TOF mass analyser. LC–MS analyses were performed with HPLC, column Onyx C18, (25 x 4.6 mm), flow 3 mL/min linear gradient CH_3CN in water 0–100% (+ 0.1% HCO_2H) in 2.5 min. Optical rotation measurements were performed at λ = 589 nm (Na lamp), the compounds were solubilized in $CHCl_3$. The ball-milling experiments were performed in a planetary mill, 12 mL steel jar (50 stainless steel balls, 5 mm Ø). The synthesis of compounds **2a–c**, **3a**, **4**, **5a** and **6** (Table 3) was performed according to previously reported procedures [1]. Their identity was assessed by comparison with the spectral data previously published [1]. The optical power was also measured: **2a** $[\alpha]_D^{20}$ – 4.11 (*c* = 1.02, $CHCl_3$); **2b** $[\alpha]_D^{20}$ - 3.33 (*c* = 1.02, $CHCl_3$); **2c** $[\alpha]_D^{20}$ + 4.66 (*c* = 1.05, $CHCl_3$); **3a** $[\alpha]_D^{20}$ - 4.60 (*c* = 1.00, $CHCl_3$); **4** $[\alpha]_D^{20}$ - 7.33 (*c* = 1.01, $CHCl_3$); **5a** $[\alpha]_D^{20}$ - 3.36 (*c* = 1.01, $CHCl_3$); **6** $[\alpha]_D^{20}$ + 8.48 (*c* = 1.05, $CHCl_3$).

General procedure for the synthesis of 3,5-disubstituted hydantoins (Table 3).

Conditions A, dry grinding. (Step 1) The α-amino methyl ester hydrochloride (1.0 equiv.) and 1,1'-carbonylimidazole (CDI) (1.3 equiv.) were ground in a 12 mL stainless steel milling jar in a planetary ball-mill at 450 rpm for 40 minutes. (Step 2) The amine (1.6 equiv) and K_2CO_3 (3.6 equiv) were added and the mixture was ground at 450 rpm for two hours. Distilled water was added to the crude and the desired compound was either precipitated and filtered over sintered glass, or extracted with ethyl acetate. The organic layer was washed with a 10% aq. citric acid solution (× 3) and brine (× 1), dried over $MgSO_4$ and concentrated in vacuo. Only for compounds **3b** and **5b** a purification by flash chromatography was performed on the crude sample, respectively.

Conditions B (with MeO-PEG-OMe, compounds 2a,b, 3a, 4, 4b and 6) and C (with HO-PEG-3400-OH, compounds 2a,b, 4, 4b and 6), wet grinding (Table 3). In the case of PEG-assisted grinding the reaction was performed by a modification of conditions A: the suitable PEG polymer (450 mg/mmol substrate) was added in Step 2. The compound was recovered by extraction with ethyl acetate after addition of distilled water to the reaction mixture. The organic layer was washed with a 10% aq. citric acid and brine, dried over $MgSO_4$ and concentrated in vacuo. When necessary, the compound was purified by flash chromatography.

***(S)*-3-[4'-(4-Methyl-1H-pyrazol-1-yl)-phenylmethyl]-5-benzyl-2,4-imidazolidinedione (3b, Table 3, entry 5).** The reaction scale was 1 mmol (conditions A). The compound was obtained by precipitation in water and purified by flash chromatography (linear gradient of AcOEt in Et_2O: 0–10%). White solid (108

mg, 30% isolated yield); $[\alpha]_D^{20}$ - 66.6 (c = 1.04, $CHCl_3$); ^{1}H NMR ($CDCl_3$, 300 MHz) δ (ppm): 7.68 (s, 1H, ImH), 7.58 (s, 1H, ImH), 7.54 (d, J = 6.2 *Hz*, 2H), 7.32 (d, J = 8.5 *Hz*, 2H), 7.32-7.23 (m, 3H, ArH), 7.20-7.12 (m, 2H,), 5.38 (s, 1H, NH), 4.60 (m, 2H, NCH_2), 4.28 (dd, J = 8.4 and J = 3.7 *Hz*, 1H, NCH), 3.27 (dd, J = 8.6 and J = 3.7 *Hz*, 1H CH_2/H_a), 2.86 (dd, $1H_b$ J = 8.6 and J = 5.4 Hz, 1H CH_2/H_b), 2.16 (s, 3H CH_3); ^{13}C NMR ($CDCl_3$ 75 MHz) δ (ppm): 172.8, 156.7, 142.1, 139.9, 135.1, 133.6, 129.7, 129.4, 129.1, 127.6, 125.4, 118.9, 118.5, 58.5, 41.7, 37.9, 29.8, 9.1; ESI-(+) *m/z*: 402.1 $[M + CH_3CN + H]^+$ 361.2 $[M + H]^+$, 268.4 $[M – PhCH_3]^+$, 228.4, 184.4, 151.9, 130.3, 100.6 $[M – CH_2PhPyr\text{-}CH_2Ph]^+$. HRMS ESI-(+): calcd for $C_{21}H_{21}N_4O_2$ $[M + H]^+$ 361.1665, found 361.1665

(*S*)-3-(Furan-2-ylmethyl)-5-benzyl-2,4-imidazolidinedione (3c, Table 3, entry 6). The reaction scale was 1.0 mmol (conditions A). The compound was obtained by precipitation in water. White solid (190.0 mg, 70% isolated yield);); $[\alpha]_D^{20}$ - 3.4 (c = 1.03, $CHCl_3$); ^{1}H NMR (DMSO-d_6, 400 MHz) δ (ppm): 8.31 (s, 1H, FuH), 7.48 (s, 1H, FuH), 7.26-7.19 (m, 3H, ArH), 7.15-7.10 (m, 2H, ArH) 6.29 (dd, J = 3.1 and J = 1.9 *Hz*, 1H, FuH), 5.83 (d, J = 3.2 *Hz*, 1H, NH), 4.45 (t, J = 5.0 *Hz*, 1H, NCH), 4.35 (q, J = 9.4 *Hz*, 2H, NCH_2), 2.97-2.95 (m, 2H, CH_2); ^{13}C NMR (DMSO-d_6, 100 MHz) δ (ppm): 173.1, 155.9, 149.5, 142.6, 135.4, 129.9, 128.3, 126.9, 110.6, 107.6, 57.3, 36.5, 34.5; ESI-(+) *m/z*: 271.2 $[M + H]^+$, 203.0 $[M - Fu]^+$, 186.3, 102.0 $[M – CHFu\text{- }CHPh]^+$. HRMS ESI-(+): calcd for $C_{15}H_{15}N_2O_3$ $[M + H]^+$ 271.1087, found 271.1083.

***(S)*-3-[4'-(4-Methyl-1*H*-pyrazol-1-yl)-phenylmethyl]-5-((carboxybenzylamino)butyl)-2,4-imidazolidinedione (5b, Table 3, entry 9).** The reaction scale was 0.7557 mmol. The crude was precipitated by AcOEt (conditions A) or purified by column chromatography (37% ^{1}H NMR yield, conditions C, linear

gradient of EtOH in Et_2O: 0-10%). White solid (223.0 mg, 62% isolated yield for Conditions A); $[\alpha]_D^{20}$ - 5.4 (c = 1.00, $CHCl_3$); ^{1}H NMR (DMSO-d_6, 300 MHz) δ (ppm): 8.36 (s, 1H, PyrH), 8.21 (s, 1H, PyrH), 7.74 (d, J = 8.4 *Hz,* 2H, ArH), 7.53 (s, 1H,), 7.41-7.18 (m, 8H, ArH + NH), 4.99 (s, 2H, CH_2O), 4.53 (s, 2H, NCH_2), 4.12 (t, J = 6.6 *Hz,* 1H, NCH), 2,96 (m, 2H, NCH_2) 2.09 (s, 3H CH_3), 1.80-1.18 (m, 6H, $(CH_2)_3$); ^{13}C NMR (DMSO-d_6, 75 MHz) δ (ppm): 174.2, 156.6, 156.1, 141.6, 139.0, 137.3, 134.2, 128.5, 128.3, 127.7, 126.0, 117.9, 117.7, 65.1, 56.3, 30.9, 28.9, 21.5, 8.71; ESI-(+) *m/z*: 476.0 $[M + H]^+$. HRMS ESI-(+): calcd for $C_{26}H_{30}N_5O_4$ $[M + H]^+$ 476.2298, found 476.2294.

(*S*)-3-(Furan-2-ylmethyl)-5-((carboxybenzylamino)butyl)-2,4-imidazolidinedione (5c, Table 3, entry 10). The reaction scale was 0.7557 mmol (conditions A). NaCl (250 mg) was added during Step 1 [2]. The crude was precipitated by Et_2O then purified by column chromatography (linear gradient of MeOH in CH_2Cl_2: 0-1%). White solid (107 mg, 37% isolated yield); $[\alpha]_D^{20}$ -26.5 (c = 1.00, $CHCl_3$); ^{1}H NMR ($CDCl_3$, 400 MHz) δ (ppm): 7.36-7.32 (m, 6H), 6.31 (m, 2H), 6.16 (m, 1H), 5.09 (s, 2H), 4.69 (sl, 1H), 4.65 (s, 2H), 4.02-3.97 (m, 1H), 3.17 (m, 2H), 1.91-1.86 (m, 1H), 1.52-1.28 (m, 5H); ^{13}C NMR ($CDCl_3$, 100 MHz): δ (ppm): 173.5, 157.0, 156.8, 156.7, 149.0, 142.6, 136.6, 128.7, 128.3, 128.2, 110.6, 108.9, 66.9, 57.1, 40.5, 40.4, 35.0, 31.1, 29.5, 21.5; ESI-(+) *m/z* 408.2 $[M+Na]^+$, 171.0 $[(M\text{-}ZNH\text{-}Fu) + H]^+$; HRMS ESI-(+) calcd for $C_{20}H_{24}N_3O_5$ $[M+H]^+$ 386.1716 found 386.1714.

***(S)*-3-(1'-Methyl-1*H*-pyrazol-3'-methyl)5-((carboxybenzylamino)butyl)-2,4-imidazolidinedione (5d, Table 3, entry 11)**: The reaction scale was 0.7557 mmol (Conditions A). The compound was recovered after liquid-liquid extraction with ethyl acetate and water. White solid (140.0 mg, 47% isolated yield); $[\alpha]_D^{20}$ - 7.64 (c = 1.02,

$CHCl_3$); ^1H NMR ($CDCl_3$, 300 MHz) δ (ppm): 7.38-7.30 (m, 5H, ArH), 7.22 (d, *J* = 2.2 *Hz,* 1H, ImH), 6.16 (d, *J* = 2.2 *Hz,* 1H), 5.84 (s, 1H, NH), 5.09 (s, 2H, CH_2O), 4.84 (m, 1H, NH), 4.67 (s, 2H, NCH_2), 4.03 (t, *J* = 5.1 *Hz,* 1H, NCH), 3.81 (s, 3H, CH_3), 3.25-3.11 (m, 2H, NCH_2), 1.98-1.82 (m, 1H, CH_2/H_a), 1.81-1.66 (m, 1H, CH_2/H_b), 1.56-1.30 (m, 4H, $(CH_2)_2$); ^{13}C NMR ($CDCl_3$, 75 MHz) δ (ppm): 173.7, 157.3, 156.7, 147.2, 136.7, 131.2, 128.7, 128.3, 104.9, 66.9, 57.1, 40.5, 39.9, 36.1, 31.1, 29.6, 21.6; ESI-(+) *m/z*: 422.1 $[M+Na]^+$, 400.2 $[M + H]^+$, 338.4, 292.1 $[M – PhCH_2O]^+$; HRMS ESI-(+): calcd for $C_{20}H_{26}N_5O_4$ $[M + H]^+$ 400.1985, found 400.1983.

References

[1] Konnert, L.; Dimassi, M.; Gonnet, L.; Lamaty, F.; Martinez, J.; Colacino, E., *RSC Advances* **2016,** *6*, 36978–36986.

[2] Konnert, L.; Gauliard, A.; Lamaty, F.; Martinez, J.; Colacino, E., *ACS Sustainable Chem. Eng.* **2013,** *1*, 1186−1191.

23-lapp-f11-180716
ml 8
proton10ppmSI CDCl3 /opt/topspin lapp-f11 23

Compound 2b

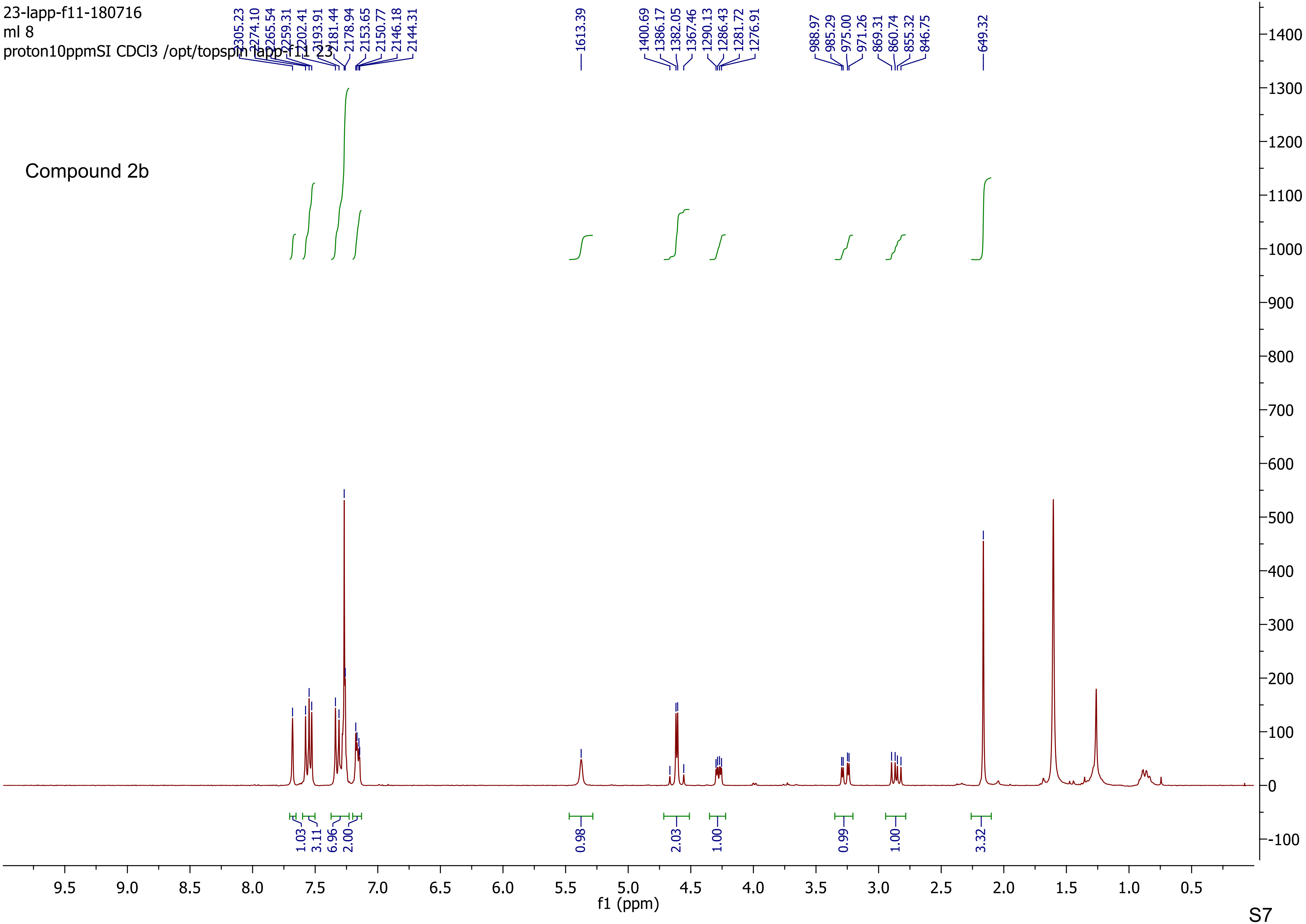

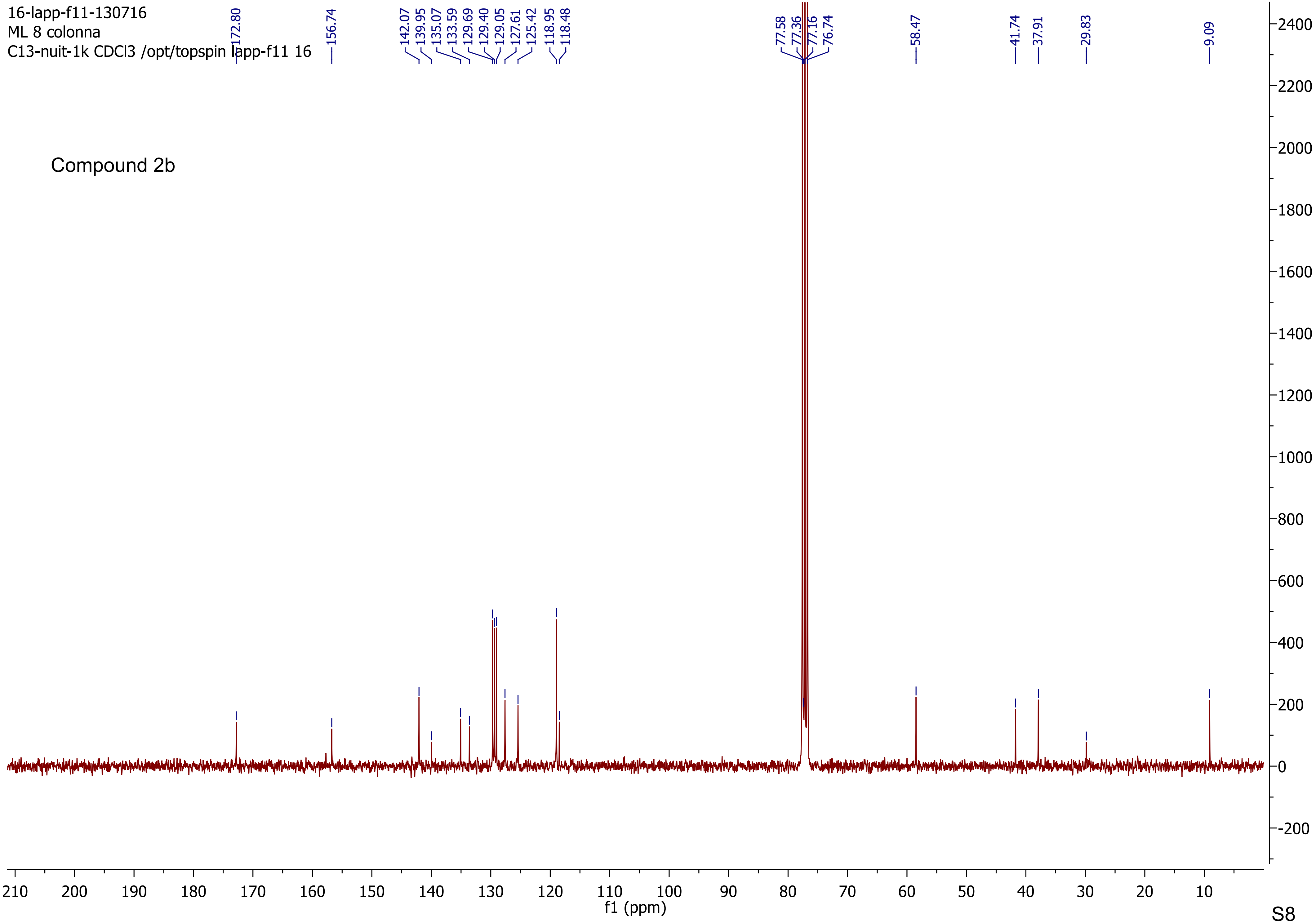
16-lapp-f11-130716
ML 8 colonna
C13-nuit-1k CDCl3 /opt/topspin lapp-f11 16
Compound 2b
172.80
156.74
142.07
139.95
135.07
133.59
129.69
129.40
129.05
127.61
125.42
118.95
118.48
77.58
77.36
77.16
76.74
58.47
41.74
37.91
29.83
9.09
f1 (ppm)

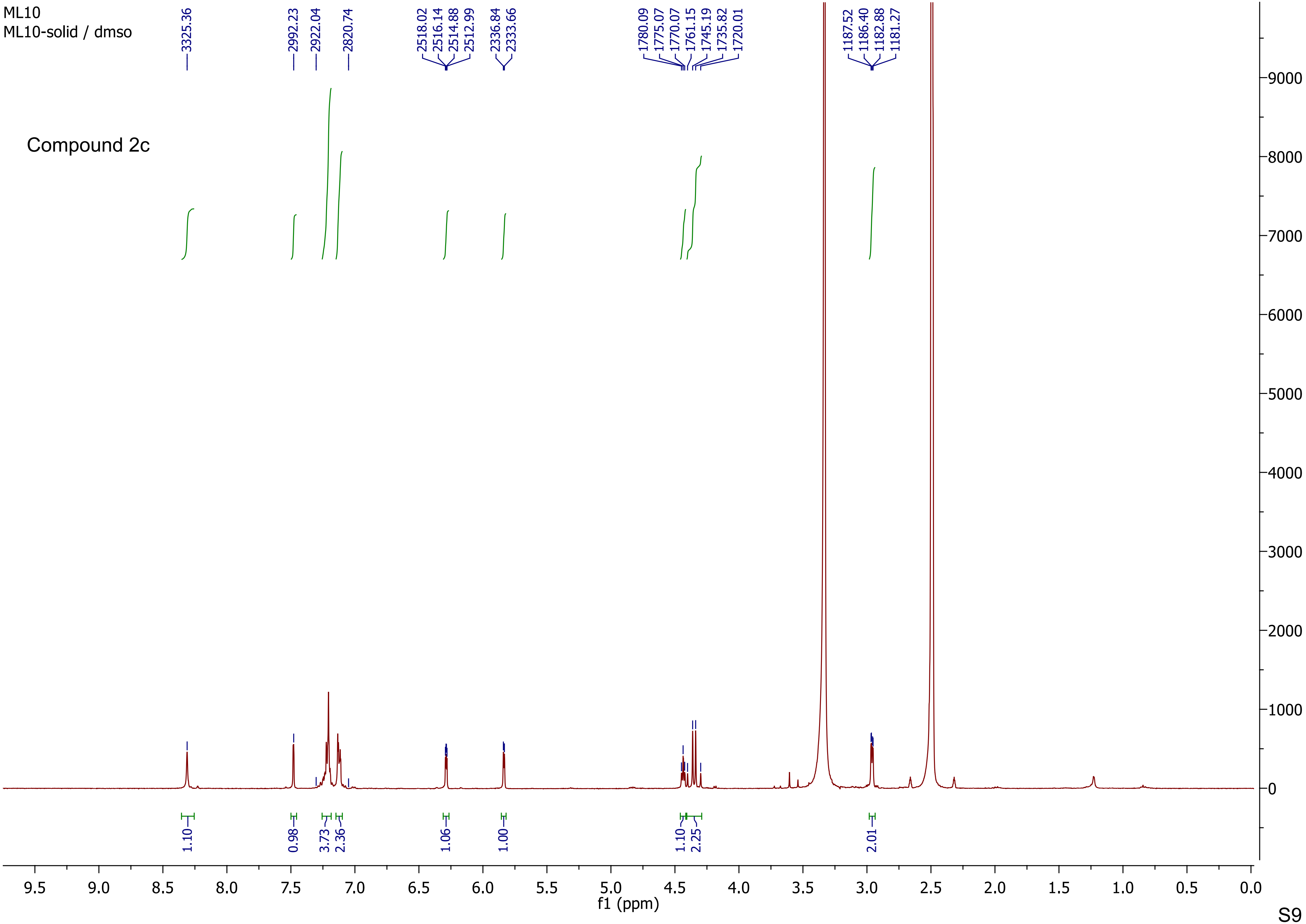
ML10
ML10-solid / dmso
Compound 2c
3325.36
2992.23
2922.04
2820.74
2518.02
2516.14
2514.88
2512.99
2336.84
2333.66
1780.09
1775.07
1770.07
1761.15
1745.19
1735.82
1720.01
1187.52
1186.40
1182.88
1181.27
1.10
0.98
3.73
2.36
1.06
1.00
1.10
2.25
2.01
f1 (ppm)

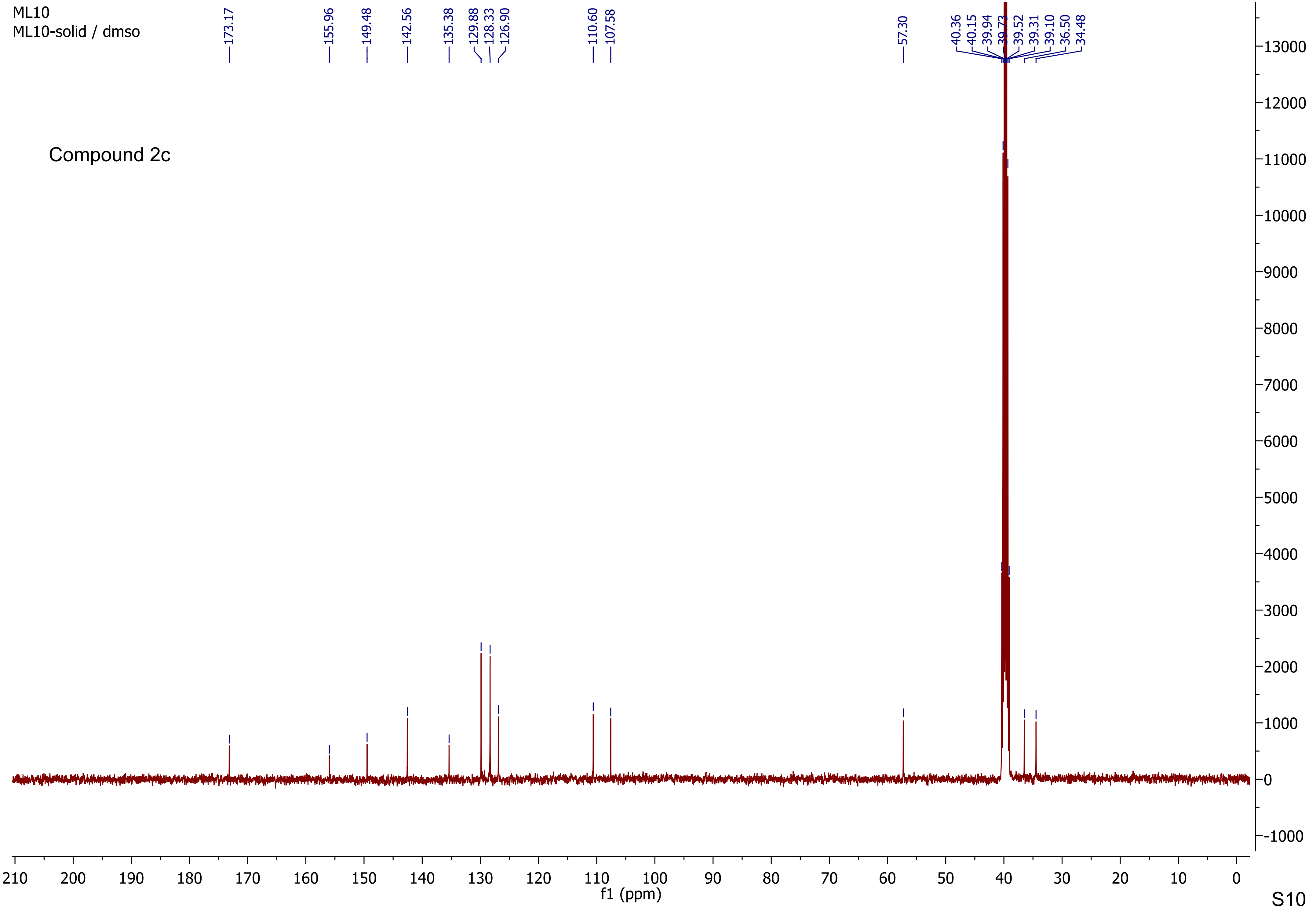
ML10
ML10-solid / dmso
Compound 2c
173.17
155.96
149.48
142.56
135.38
129.88
128.33
126.90
110.60
107.58
57.30
40.36
40.15
39.94
39.73
39.52
39.31
39.10
36.50
34.48
f1 (ppm)

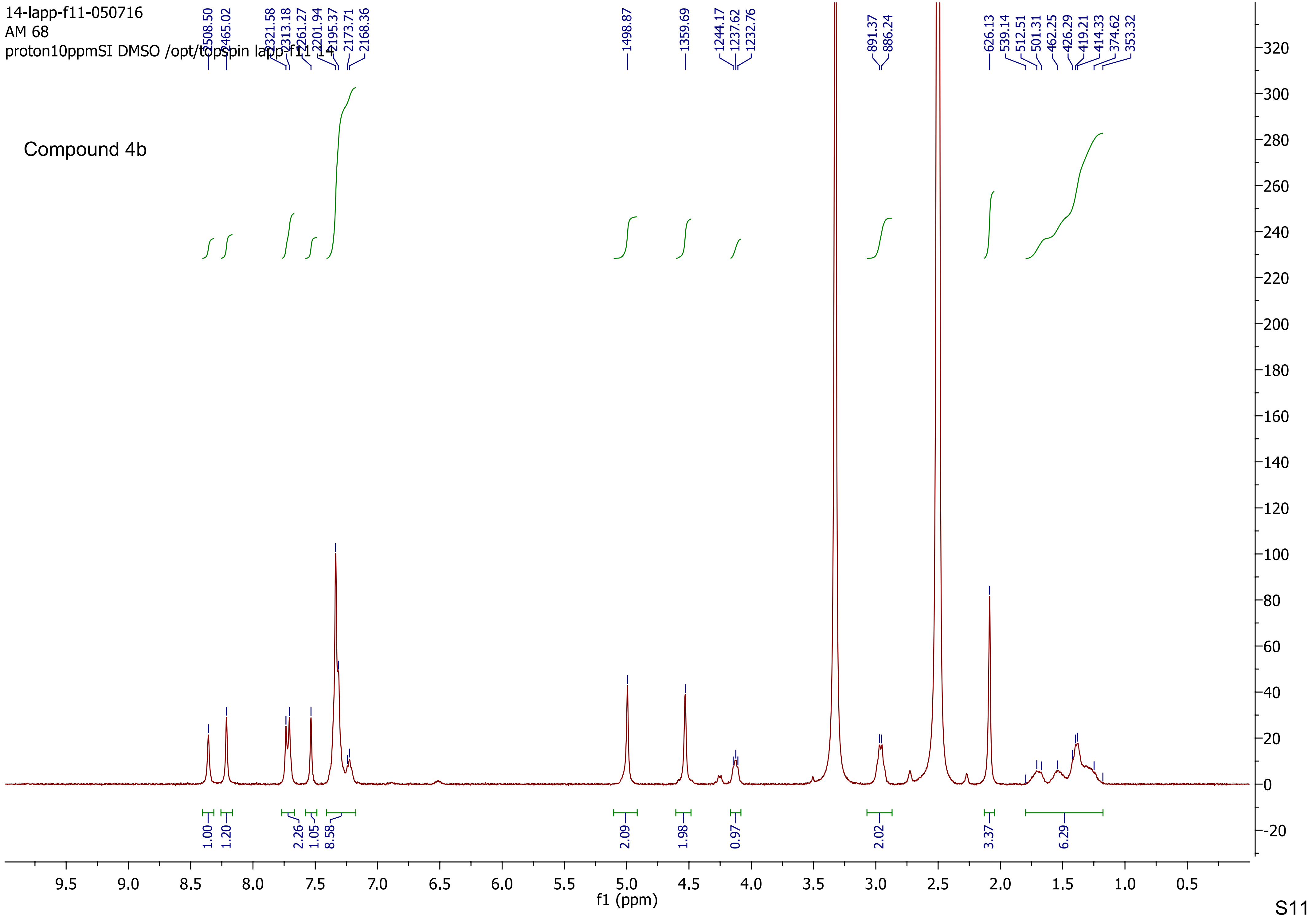
14-lapp-f11-050716
AM 68
proton10ppmSI DMSO /opt/topspin lapp-f11 14
Compound 4b
2508.50
2465.02
2321.58
2313.18
2261.27
2201.94
2195.37
2173.71
2168.36
1498.87
1359.69
1244.17
1237.62
1232.76
891.37
886.24
626.13
539.14
512.51
501.31
462.25
426.29
419.21
414.33
374.62
353.32
1.00
1.20
2.26
1.05
8.58
2.09
1.98
0.97
2.02
3.37
6.29
f1 (ppm)

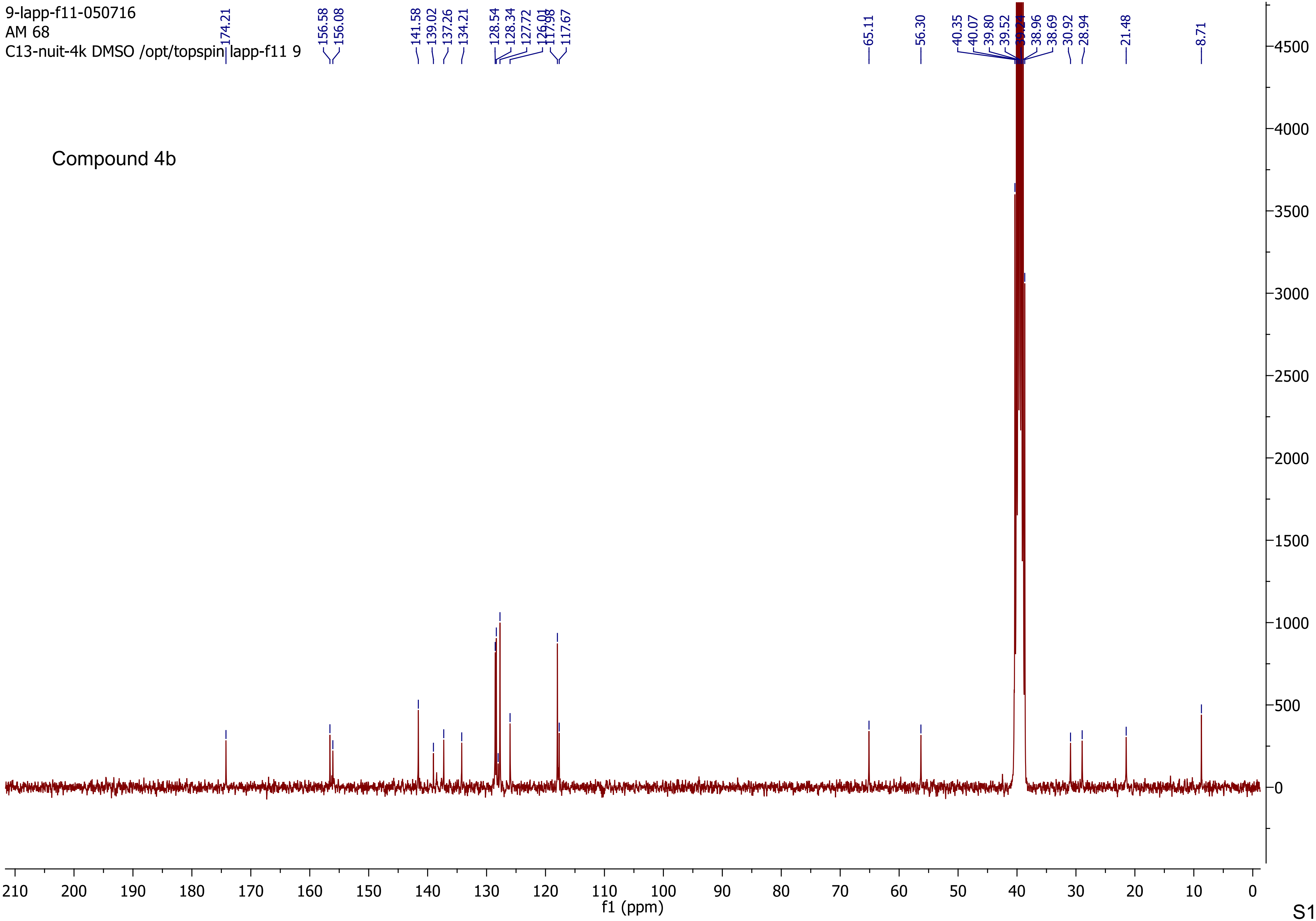
9-lapp-f11-050716
AM 68
C13-nuit-4k DMSO /opt/topspin lapp-f11 9
Compound 4b
174.21
156.58
156.08
141.58
139.02
137.26
134.21
128.54
128.34
127.72
126.01
117.98
117.67
65.11
56.30
40.35
40.07
39.80
39.52
39.24
38.96
38.69
30.92
28.94
21.48
8.71
f1 (ppm)

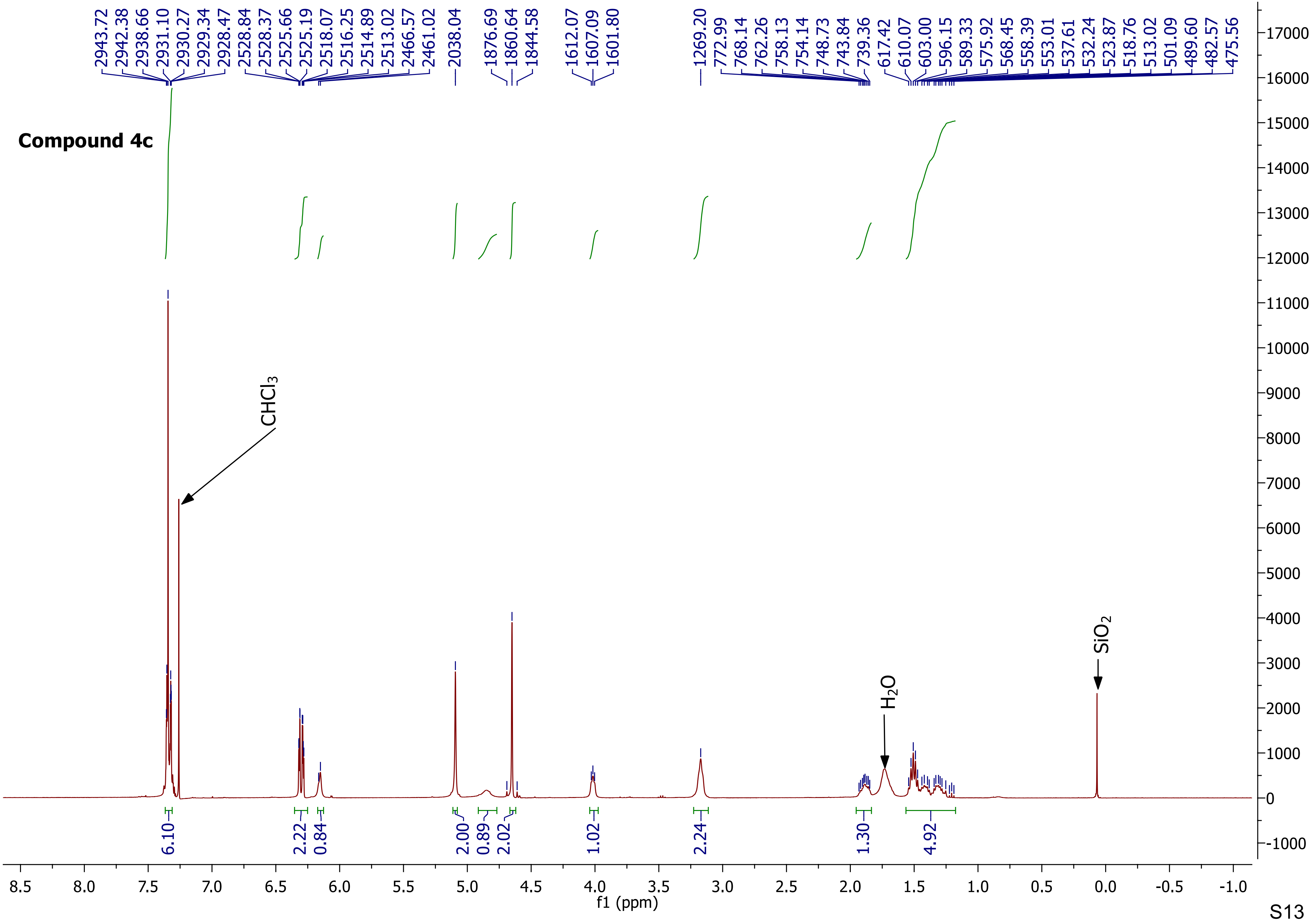
Compound 4c
2943.72
2942.38
2938.66
2931.10
2930.27
2929.34
2928.47
2528.84
2528.37
2525.66
2525.19
2518.07
2516.25
2514.89
2513.02
2466.57
2461.02
2038.04
1876.69
1860.64
1844.58
1612.07
1607.09
1601.80
1269.20
772.99
768.14
762.26
758.13
754.14
748.73
743.84
739.36
617.42
610.07
603.00
596.15
589.33
575.92
568.45
558.39
553.01
537.61
532.24
523.87
518.76
513.02
501.09
489.60
482.57
475.56
CHCl3
H2O
SiO2
6.10
2.22
0.84
2.00
0.89
2.02
1.02
2.24
1.30
4.92
f1 (ppm)

Compound 4c

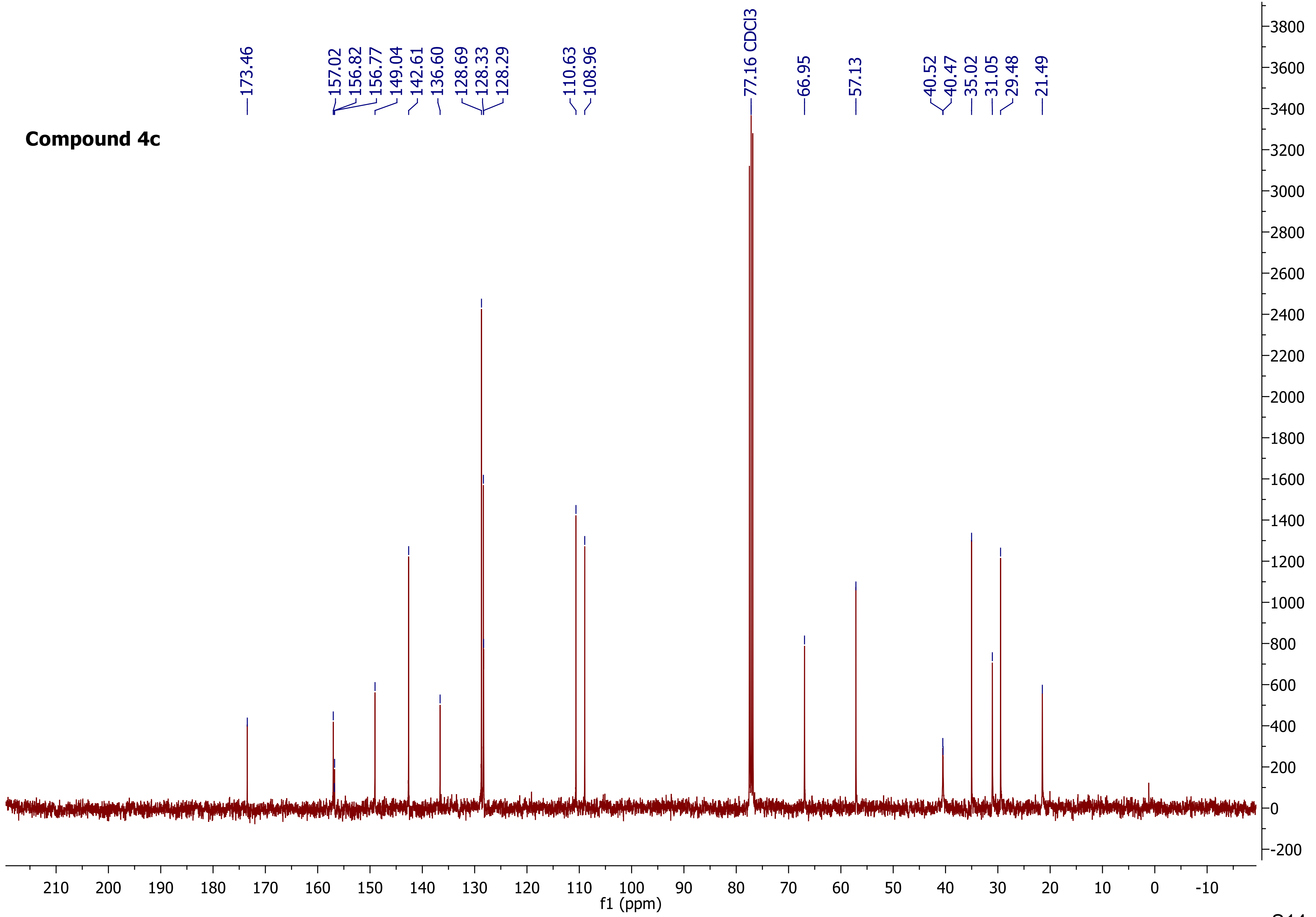

173.46
157.02
156.82
156.77
149.04
142.61
136.60
128.69
128.33
128.29
110.63
108.96
77.16 CDCl3
66.95
57.13
40.52
40.47
35.02
31.05
29.48
21.49
f1 (ppm)

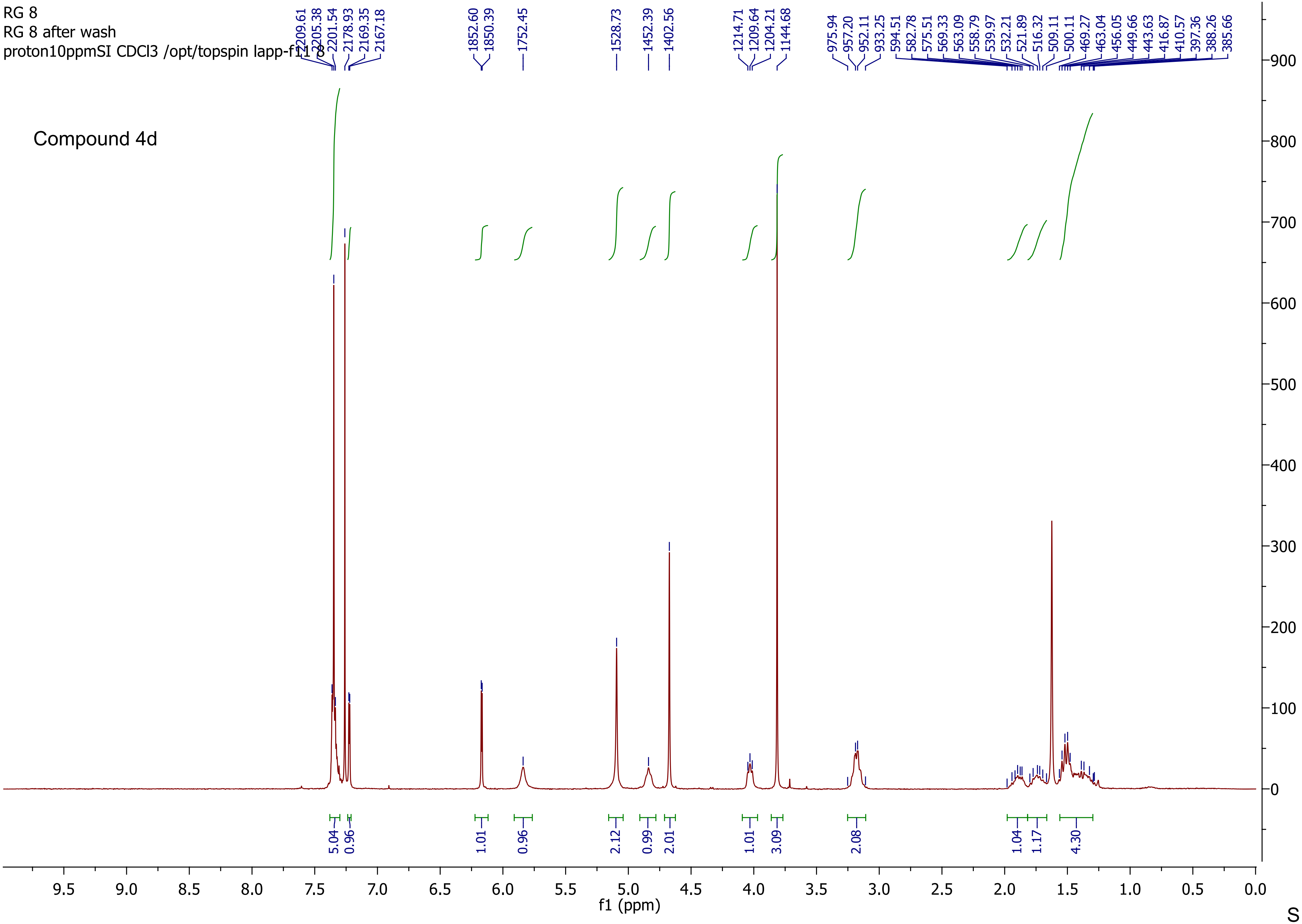
RG 8
RG 8 after wash
proton10ppmSI CDCl3 /opt/topspin lapp-f11 8
Compound 4d
2209.61
2205.38
2201.54
2178.93
2169.35
2167.18
1852.60
1850.39
1752.45
1528.73
1452.39
1402.56
1214.71
1209.64
1204.21
1144.68
975.94
957.20
952.11
933.25
594.51
582.78
575.51
569.33
563.09
558.79
539.97
532.21
521.89
516.32
509.11
500.11
469.27
463.04
456.05
449.66
443.63
416.87
410.57
397.36
388.26
385.66
5.04
0.96
1.01
0.96
2.12
0.99
2.01
1.01
3.09
2.08
1.04
1.17
4.30
f1 (ppm)

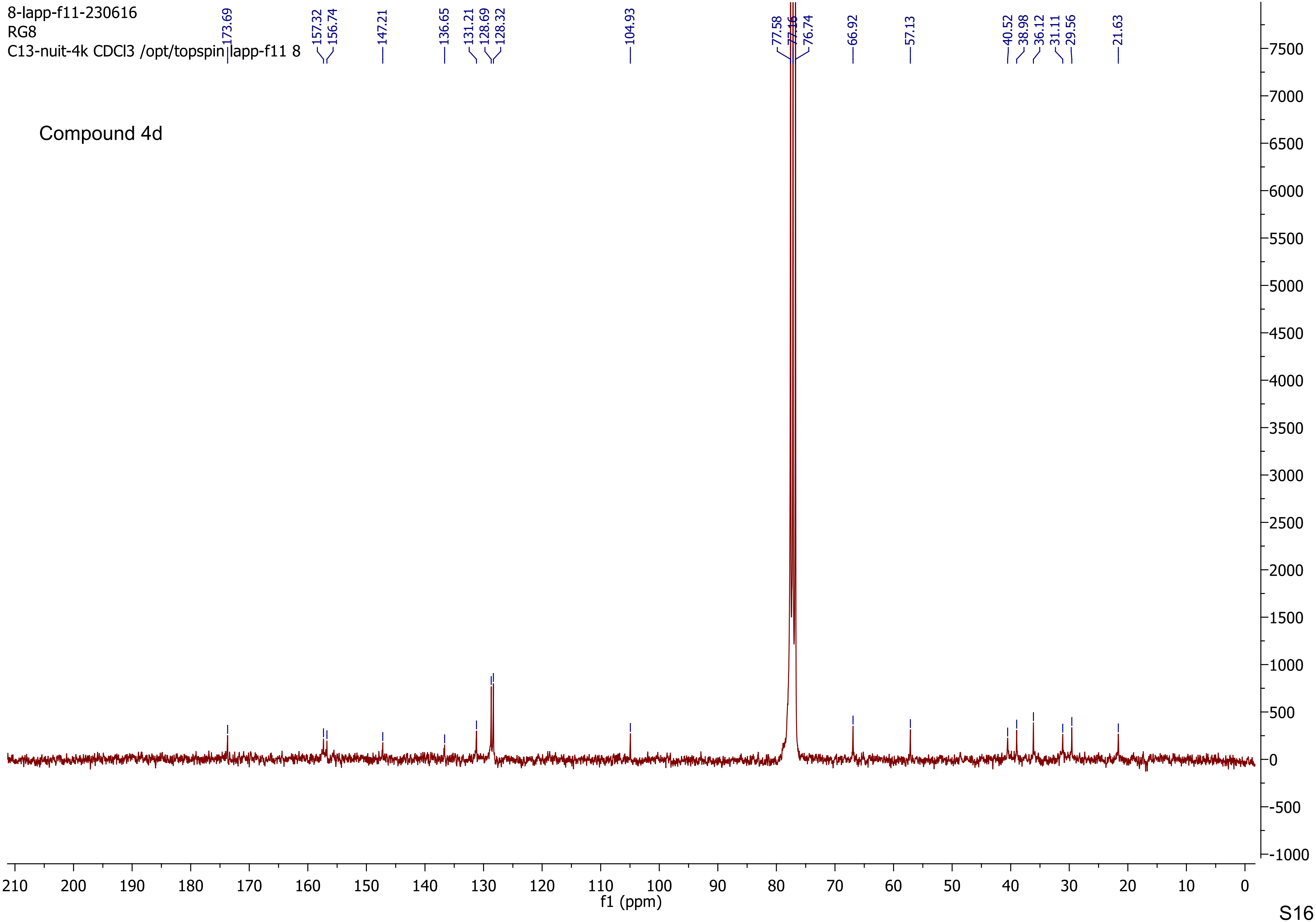

8-lapp-f11-230616
RG8
C13-nuit-4k CDCl3 /opt/topspin lapp-f11 8
Compound 4d
173.69
157.32
156.74
147.21
136.65
131.21
128.69
128.32
104.93
77.58
77.16
76.74
66.92
57.13
40.52
38.98
36.12
31.11
29.56
21.63
f1 (ppm)
